# Differences in Neurocognitive Development Between Children Who Had Had No Breast Milk and Those Who Had Had Breast Milk for at Least 6 Months

**DOI:** 10.3390/nu17172847

**Published:** 2025-08-31

**Authors:** Neil Goulding, Kate Northstone, Caroline M. Taylor, Pauline Emmett, Yasmin Iles-Caven, Jacqueline Gregory, Steven Gregory, Jean Golding

**Affiliations:** 1 Centre for Academic Child Health, Population Health Sciences, Bristol Medical School, University of Bristol, Canynge Hall, 39 Whatley Road, Bristol BS8 2PS, UK; n.goulding@bristol.ac.uk (N.G.); caroline.m.taylor@bristol.ac.uk (C.M.T.);; 2 Population Health Sciences, Bristol Medical School, Children of the 90s, Learning & Research Building, Southmead Hospital, Bristol BS10 5NB, UK; 3 Bristol Veterinary School, University of Bristol, Bristol BS40 5DU, UK

**Keywords:** ALSPAC, breast feeding, cognition, behaviour, IQ, educational attainment, attention, speech, personality, handedness, pragmatic conversation

## Abstract

**Background**: There is considerable evidence that breast feeding has a beneficial effect on the neurocognition of a child. However, most studies have confined their attention to the Intelligence Quotient (IQ), tending to ignore other aspects of neurodevelopment. **Methodology**: Here we present the relationship between breast feeding for at least 6 months with 373 neurocognitive outcomes measured from infancy through to late adolescence using data collected in the Avon Longitudinal Study of Parents and Children (ALSPAC). We first examined unadjusted regression associations with breast feeding at age 6 months. Where the unadjusted *p*-value was < 0.0001 (*n* = 152 outcomes), we adjusted for social and other factors. **Results**: This resulted in 42 outcomes with adjusted associations at *p* < 0.001. Specifically, these included associations with full-scale IQ at ages 8 and 15 years (adjusted mean differences [95% confidence interval (CI)] +4.11 [95% CI 2.83, 5.39] and +5.12 [95% CI 3.57, 6.67] IQ points, respectively, compared to not breastfeeding for 6 months). As well as the components of IQ, the other phenotypes that were strongly related to breast feeding for at least 6 months were measures of academic ability (reading, use of the English language and mathematics). In accordance with the literature, we show that children who are breast fed are more likely to be right-handed. The one association that has not been recorded before concerned aspects of pragmatic speech at 9 years where the children who had been breast fed were shown to perform more appropriately. **Conclusions**: We conclude that breast feeding for at least 6 months has beneficial effects on a number of neurocognitive outcomes that are likely to play a major part in the offspring’s future life course. We point out, however, the possibility that by using such stringent *p*-value criteria, other valid associations may have been ignored.

## 1. Introduction

There have been many studies concerning the neurocognitive benefits to the child who had been breast fed compared to children who had received no breast milk, with inconsistent results, with some showing positive and occasionally negative associations, often as the result of small sample sizes and tests that were not comparable. Nevertheless, there has been convincing evidence, particularly from the PROBIT clustered RCT of advice concerning exclusive breast feeding to mothers in Belarus where follow up of the children to age 6.5 years showed that the children born at hospitals randomised to the intervention had increased vocabulary, verbal IQ, and similarities, and the teachers rated their reading and writing as better than children born at hospitals without the intervention [1]. The PROBIT children were tested again at age 16 and showed increases in memory and verbal function in the intervention group compared with the control group [2]. Conversely no differences were found between the two groups regarding the children’s behaviours [3].

Systematic reviews of the association between breast feeding and IQ have also shown evidence of positive associations [4]. Other systematic reviews found small but positive associations with cognition, behaviour and executive function [5]. Hou and colleagues used a ratio of means analysis and showed positive associations with IQ when comparing any breast feeding with none, and with breast feeding of over 6 months compared with less than or equal to 6 months [6].

Large observational studies that have used other neurocognitive outcomes include a study of 177,000 children in Scotland which showed that those children who had been exclusively breast fed for 6–8 weeks had a lower risk of having Special Educational Needs due to learning difficulties when compared with those who had had no breast milk; however, the children who were breast fed for 6–8 weeks, but not exclusively, were as unlikely to have such an outcome as were the children who had been exclusively breast fed [7]. In Japan, a longitudinal study of 77,000 children found that breast feeding for at least 6 months resulted in a lower risk of developmental delay at 12 months; the authors also showed similar results using pairs of siblings [8]. An Australian Longitudinal Study of 8560 children showed positive associations between the duration of breast feeding with language skills and non-verbal intelligence but not with executive function [9].

Thus, the literature indicates repeated support for an association between breast feeding and IQ, but there have been few studies showing consistent findings with other neurocognitive outcomes using large datasets. The aim of this set of analyses is to take advantage of the wealth of neurocognitive outcomes recorded on the children taking part in the Avon Longitudinal Study of Parents and Children (ALSPAC) to determine the types of neurocognitive outcomes that differ between children who were breast fed for at least 6 months and those who were not breast fed at all.

## 2. Materials and Methods

### 2.1. The ALSPAC Pre-Birth Cohort

In April 1990, in the English county of Avon, the Avon Longitudinal Study of Parents and Children (ALSPAC), a pre-pregnancy longitudinal study, began with the aim of identifying the factors (both environmental and genetic) that influence a child’s health and well-being [10]. The study was designed to enrol all pregnant women resident in the defined area with an expected date of delivery between 1 April 1991 and 31 December 1992 inclusive. Approximately 75–80% (*n* = 14,541) of eligible women joined the study prior to or immediately after birth [11,12].

Data were collected using self-completed questionnaires, posted directly to the mothers. A questionnaire for her partner (with a reply-paid envelope) was sent to the mother to hand to her partner if she so wished. This arrangement was made on the advice of the study’s Ethics Committee (see [13] for further details of the discussions and decisions made).

Ethical approval for the study was also obtained from the ALSPAC Ethics and Law Committee and the Local NHS Research Ethics Committees. Implied consent from participants for the use of data collected via questionnaires was assumed following the recommendations of the ALSPAC Ethics and Law Committee (ALEC) at the time [13]. ALEC has remained independent of ALSPAC throughout and has been approved by the American Institutional Review Board (IRB no.00003312). Detailed information on the ways in which confidentiality of the cohort is maintained may be found on the study website: http://www.bristol.ac.uk/alspac/researchers/research-ethics/ (accessed on 1 January 2025).

Please note that the ALSPAC website contains details of all the data that are available through a fully searchable data dictionary and variable search tool: http://www.bristol.ac.uk/alspac/researchers/our-data/ (accessed on 1 January 2025).

### 2.2. Details of Breast Feeding

Mothers were sent questionnaires in which details of infant feeding were obtained when the children were aged 4 weeks, 6 months and 15 months. Although details of other foods given to the baby were collected, for this study we do not use any dietary data other than whether the mother was breast feeding at the time the 6-month questionnaire was completed. Thus, we do not include any other nutrients as confounders, nor do we distinguish between those exclusively and non-exclusively breast fed.

### 2.3. Confounders

Traditionally, factors that are related to both the independent and dependent factors are chosen as confounders. For this study, since there were a large number of different outcomes, each of which may have been associated with different exposure variables, for consistency we chose to concentrate on factors that were associated with breast feeding and used those for all adjusted analyses. The following were therefore used as confounders: Maternal education level achieved (5-point scale from No qualifications to University degree); Paternal education (using similar scale); Maternal age at time of birth of child,; whether the child was first-born or not; tenure of their home (owned/mortgaged v. rented/other) is included as a marker of social (dis)advantage; delivered by Caesarean section; Mother smoked at 18 weeks of pregnancy. The reason for these choices were that, in Britain: (i/ii) parental education levels are strongly related to choosing to breast feed; (iii) Age of the mother at birth of the child since young ages are associated with failure to breast feed successfully; (iv) whether the child was first born is important since the mother is less likely to breast feed successfully with her first-born; (v) tenure of the home is included as it is a strong marker of social (dis)advantage, with those mothers living in rented accommodation being far less likely to breast feed successfully; (vi) prolonged breast feeding is less likely after delivery by Caesarean section [14]; (vii) Maternal prenatal smoking since it is associated with reluctance to breast feed as well as lower levels of cognition in the child [15,16].

### 2.4. Outcome Measures

The ALSPAC neurocognitive data include measurements, mainly using scales, some of which were completed by the mother, others by the child’s teachers, and some by the children themselves. Additional in person studies were completed by trained ALSPAC staff either overseeing completion of tests/scales on-line by the child or by direct examination. To maximize statistical power, we concentrated on the scales which were continuous, of which 373 were available for detailed analysis.

### 2.5. Statistical Analyses

Since there were a huge number of outcomes to consider, to minimise both the Type I and Type II error rates, we started by selecting for further analysis only the scales for which the unadjusted association with breast feeding at 6 months was at *p* < 0.0001. This resulted in confining the analyses to 152 of the 373 outcomes. When considering neurocognitive outcomes we confined the analyses to outcomes ascertained beyond 6 months.

Initial adjusted analyses took account of the first six confounders outlined above (Model 1). Further adjustment was then made for maternal smoking at 18 weeks gestation (Model 2). In general, since each outcome was measured on a continuous scale, multivariable linear regression was employed, using the raw scores of each outcome, and taking account of the confounders. The details of effect sizes for each outcome that resulted in *p* < 0.001 after adjustment are highlighted in the tables.

### 2.6. Missing Data

We have not modelled using missing data techniques as the data are unlikely to be missing at random. The proportion of missing data varies for each variable, but can be readily ascertained from the ALSPAC study data dictionary (www.bristol.ac.uk/alspac/researchers/our-data, accessed on 1 January 2025); it varies from 0% for the sex of the child to 62% for the IQ measure at age 15.

## 3. Results

### 3.1. Breast Feeding Prevalence

In total 11,337 mothers completed the question when the child was 6 months old concerning whether he/she was being breast fed: 28.7% were still being breast fed; 24.4% had never been breast fed and 46.9% had started but stopped breast feeding before the child was six months of age. Among those breast feeding at 6 months the frequency of feeds varied from one to 10 times a day, probably reflecting the fact that many women were still feeding on demand. We use all children who were receiving breast milk at 6 months as the focus of this set of analyses which assesses the ways in which their subsequent development differed from children who had never received breast milk. Those children who received breast milk for less than 6 months are omitted from the analyses.

### 3.2. Child Development

During the pre-school period there were two scales that were used to assess child development: the Griffiths tests [17], which were administered to the children at 18 months by trained psychologists—these assessed five attributes (locomotor, social/personal, hearing/speech, hand/eye coordination, and performance) together with a total score. Of these six scales, prior to adjustment, three were positively associated at *p* < 0.0001 with breast feeding at 6 months (social/personal, hearing/speech and the total score). After adjustment, although the associations were still positive, none were associated at *p* < 0.001 (Table 1).

A similar set of scales had been developed for completion by the child’s main carer (usually the mother) based on the Denver screening inventories [18]. These were asked at 18, 30, 42 and 57 months and comprised scales for social skills, fine motor ability, communication skills, gross motor skills and then a total score. Of the 25 scales tested, six of the associated associations with breast feeding were at *p* < 0.0001 (Table 1), but only two survived adjustment at *p* < 0.001, and both were related to fine motor skills. Note that none of the unadjusted age-related Denver total development scores were associated at *p* < 0.0001.

Thus, of the 31 preschool developmental assessments using our stringent *p*-value criteria, the only associations that survived adjustment concerned fine motor skills at 30 and 42 months of age.

### 3.3. Measures of Cognition Using IQ Scales

Tests using the scales from the Weschler group of tests were undertaken in a standardised setting by trained ALSPAC staff (mostly psychologists). At age four years, a 10% representative subsample known as the Children in Focus were administered the Wechsler Preschool and Primary Scale of Intelligence [19]. At 8 years, the whole sample were invited to attend for an abbreviated form of the UK version of the Wechsler Intelligence Scale for Children (WISC) [20], for which alternative items of each scale were used. At 15 years, all original offspring were invited for a further IQ test—The Wechsler Abbreviated Scale of Intelligence (WASI-II) [21] which was designed for individuals between 6 and 90 years of age. Because of shortage of finance at the time, only two of the four subtests were administered: the verbal comprehension index and the perceptual reasoning index. The sum of these resulted in the total IQ.

All unadjusted associations were positive at *p* < 0.0001. In addition, except for the 4-year tests where numbers for adjusted analyses were small (≤450), all adjusted analyses remained positive at *p* < 0.001. This was true of verbal, performance and total IQ measures as well as for specific subtests (Table 2).

### 3.4. Memory

At 8 years of age, the digit span test, using both forward and backward formats, was administered to the children as part of the WISC test of IQ [20]. It measures both short-term and working memory. At the same 8-year assessment, an adaptation of the Nonword Repetition Test [22] was used to assess the children’s working memory. This comprised twelve nonsense words, four each of 3, 4 and 5 syllables and conforming to English rules for sound combinations. The child was asked to listen to each word via an audio cassette recorder and then repeat each item. This was also used in a standardised situation at ages 9, 12 and 13 years. At age 10 years working memory was assessed using the Counting Span Task [23] which requires the simultaneous processing and storage of information as well as working memory.

Although there were strong positive unadjusted associations between all memory tests among children who had been breast fed for 6 months compared with those who had never been breastfed, after adjustment the *p*-values all exceeded 0.001 except for the non-word repetition measure at age 8 which continued to show a strong association. (Table 3).

### 3.5. Speech and Language

There were 44 tests of speech and language as the child developed, including both testing face-to-face by trained ALSPAC employees (*n* = 20 tests) and maternal responses to detailed questionnaires concerning the child’s vocabulary and grammatical skills (*n* = 24 tests). The face-to-face assessments included the verbal comprehension and alveolars subtest of the Reynell Developmental Language Scales [24] on the 10% subgroup—the Children in Focus at 25 and 61 months, and the UK version of the Weschler Objective Language Dimensions Manual (WOLD) [25] to which the whole cohort were invited at 8 years. Children breast fed for 6 months compared with those never fed breast milk showed a positive unadjusted association in the comprehension test at *p* < 0.0001 but failed to reach *p* < 0.001 on adjustment. All other tests shown in Table 4 were completed by the mother at various ages. Of note are the associations with the sets of questions that are part of the Children’s Communication Checklist (CCC) [26], many of which showed positive associations with breast feeding for 6 months, even after adjustment. This checklist was developed specifically to identify children with pragmatic speech impairments. ALSPAC coded the subtest scores so that the positive scores were all positive (good) outcomes. Thus, the positive adjusted mean differences found here for inappropriate initiation, stereotyped conversation, use of conversation, and pragmatic use of conversation, indicate more appropriate initiation of conversation, less stereotyped conversation, improved use of conversation; in general, the higher the score on pragmatic use of conversation, the better and more appropriately interactive the child’s conversation.

### 3.6. Reading, Spelling and English Language

There were 18 tests of reading ability. Just as shown with measures of IQ, most of the measures of reading ability (for description of tests see [27]) were shown to be strongly related to breast feeding at 6 months, the exception being the child’s own perception of their reading ability which was not different at *p* < 0.001 from their peers who had not had any breast milk. The increased reading ability of the breast-fed group applied to the five tests administered by the ALSPAC team between the ages of 7 and 13 years, as well as the standard test on school entry and the National tests at SATS3 (ages 15–16 years). The associations with spelling, although positive. were less convincing and failed to reach our stringent *p*-value after adjustment. National tests involving the English language and writing ability, however, both showed positive associations with breast feeding and survived adjustment at *p* < 0.001 (Table 5).

### 3.7. Mathematics and Science

Tests of arithmetic were administered to the children at ages 4 and 8; Although the unadjusted associations with breast feeding were positive at *p* < 0.0001, on adjustment neither association survived. In addition, there were three separate tests developed for ALSPAC: they were devised to identify mathematical understanding and were administered by the children’s schoolteacher. All showed positive associations with breast feeding, that administered at age ten surviving adjustment. In addition, the two National tests of mathematics both showed strong positive associations with breast feeding that survived adjustment (Table 6).

Two scientific tests were administered by the schoolteacher. They measured scientific reasoning and scientific comprehension, respectively. Although both showed positive results with breast feeding, neither result survived adjustment at *p* < 0.001 (Table 6).

### 3.8. Temperament Pre-School

The mother was given questionnaires to assess the temperament of her child. These included the Carey scales at 6 and 24 months of age each of which assessed nine different temperaments [28,29]. We have not considered the 6-month Carey measures here since the breast-feeding mother was still feeding at this time. At 3, 4 and 5 years the Emotionality Activity Sociability Temperament Survey (EAS) was used [30]. This identified four different temperaments at each age. As can be seen from Table 7, of the 21 different age/temperament combinations originally tested, eight were initially associated with breast feeding at *p* < 0.0001, but only three survived adjustment to *p* < 0.001; these comprised negative associations with activity at 3, 4 and 5 years implying that the breast fed children were less active (or less hyperactive) than those who had not had breast milk.

### 3.9. Behaviour

The major set of scales completed by the mother, the teacher and the children themselves in adolescence, was the Strengths and Difficulties Questionnaire (SDQ) [31]. This comprises six scales: prosocial behaviour, hyperactivity, peer problems, emotional symptoms and conduct problems together with total behaviour difficulties. These six scales were obtained on seven occasions. Of the 42 behaviours 19 were associated with breast feeding prior to adjustment, and only one after adjustment (a negative association with hyperactivity).

At age 7, the Developmental and Well-being Assessment (DAWBA) [32] was completed by the mother and partially by the teacher. Of the 30 unadjusted scales, only five were at *p* < 0.0001 and none survived adjustment (Table 8). Additional behaviours asked were not included in Table 8 if they did not achieve *p* < 0.0001 in the unadjusted association with breast feeding.

### 3.10. Personality and Other Attributes

In Table 9 are descriptions of the associations between breast feeding until 6 months with: (i) Locus of control (using the Nowicki scales [33] at ages 8 and 16 (the higher the score, the more extraverted); (ii) personality using the Big Five scales [34]: Extraversion, Agreeableness, Conscientiousness, Emotional stability and Intellectuality; (iii) 32 measures of Motor coordination and balance; (iv) 39 tests of attention, inhibition and executive function; (v) 12 measures related to addictions, and (vi) two of cognitive style [35]. Of these, only two survived adjustment: locus of control at age 8, where the children who had been breast fed were more internal, and a scale indicating that the breast fed were more likely to be right-handed (scale made up of the hand used for six activities (e.g., holding toothbrush, writing, holding spoon) completed at 42 months.

## 4. Discussion

### 4.1. Summary of Results

This detailed analysis of 373 neurocognitive measures, with very strict rules concerning the *p*-values at the two stages of analysis has resulted in the identification of just 42 neurocognitive traits being associated on at least two measures with being breast fed for at least six months (i.e., *p* < 0.0001 when unadjusted and *p* < 0.001 after adjustment). These can be categorised as follows: positive results for traits related to IQ, reading ability, fine motor skills pre-school, development of speech including pragmatic conversational abilities, ability in mathematics, and reduction in risk of hyperactive behaviour. Other associations for which there was only one result satisfying our restrictive *p*-values included: working memory at age 8 (but not at other ages), prosocial behaviour at 9 (but not at other ages), locus of control at 8 (but not 16).

The aim of this study was to assess whether there were unexpected associations with any of the neurocognitive measures collected on a continuous scale by ALSPAC. Although several studies have found positive associations with the developing child’s vocabulary and verbal IQ, we additionally found strong associations with conversational ability of 9-year-olds using the pragmatic subscales first developed by Bishop [26]. A search of the literature highlights the fact that there have been no other publications to date looking at this association. We hope that our finding will encourage other longitudinal studies to do so.

### 4.2. Validity of These Results

The most convincing signs of validity of our results lie in comparison with other studies, particularly with PROBIT—the major clustered RCT which took place in Belarus [1]. Our general relationships with IQ, reading and speech mirror the findings of PROBIT. However, the PROBIT RCT showed no association with child behaviour [1,2] whereas we found a reduced association with active temperament in the pre-school period, as well as of hyperactive behaviour when at school age. Nevertheless, this lower level of hyperactivity with prolonged breast feeding is similar to that found in a systematic review [36].

Although we had not predicted an association with handedness, there have been publications from Britain and Ireland [37] that had found this association to be related to duration of breast feeding, and a subsequent systematic review also found that infants who had been breast fed for 3 months or more were more likely to be right-handed at *p* < 0.0001 [38]. Thus, the majority of our main findings are supported by the literature. As noted above, however, the strong associations with measures of pragmatic conversation could not be validated in this way since no other study had addressed such an analysis.

It is convincing to know that our results in general have been confirmed by other studies—particularly the RCT in Belarus. However, the stringent *p*-values that we applied may well have resulted in the elimination of results that are valid, causal and potentially important.

### 4.3. Possible Mechanisms

There is often debate of causation of associations with breast feeding concerning whether the results are more to do with the physical and emotional closeness between the breast-feeding mother and her child rather than social or other factors. However, the well-designed comparison GUSTO study in Singapore compared (a) the development of children fed breast milk by bottle with those fed artificial milk by bottle and showed that the breast milk group had a higher IQ. Conversely, (b) they compared children fed breast milk by bottle with those naturally breast-fed—the latter were found to have an improved memory [39]. On this evidence it seems likely that breast milk ingestion is the important factor for the effect on IQ rather than contact between mother and child, although it would be more convincing if there were other similar studies to confirm the GUSTO findings.

A further explanation of our findings is likely to be the associations between the presence of specific nutrients in breast milk and their association with brain development during the infants’ early months. There is evidence that breast milk can reflect the nutrients present in the nursing mother’s diet [40]. Kim [41] and others [42] outlined the various nutrients present in breast milk and concluded that human breast milk ‘is the most important source of nutrition for infants.’. Among other nutrients mentioned [40] were fatty acids that were the precursors to the omega-3 fatty acids such as DHA (docosahexaenoic acid) and EPA (eicosapentaenoic acid) which are also components of fish which has been shown to be associated with the child’s IQ when consumed by the mother during pregnancy [42]. Other components of breast milk that are included in breast milk and have been shown to have an effect on the child’s neurocognitive function when consumed in pregnancy involve dietary vitamin B_12_ [27] suggesting that future studies should be carried out to determine whether children breast fed by mothers who consume various nutrients have neurocognitive benefits compared with children whose breast feeding mothers consume lower levels of such nutrients during the time they breast fed.

However, although in most of the studies of breast feeding effects, little attention is paid to the non-breast fed group—in particular to what type of foods or drinks they were given. These are likely to have differed over both time and place, thus making comparisons between observational studies to be difficult to interpret. An apparent beneficial effect of breast milk may be disguising a harmful effect of specific foodstuffs.

### 4.4. Strengths and Limitations

The strengths of this study are: (i) that the data are concerned with a whole geographic population, not restricted to births in a particular hospital or mothers with a particular social background; (ii) the data on cognitive background was obtained from sources that were blind to whether the child had been breast fed or not—including teacher reports on behaviours and abilities, in-house testing, and linkage to results from standard national assessment tests; (iii) the data available within ALSPAC allowed for detailed analyses taking account of appropriate confounders; (iv) other studies have controlled for maternal but not paternal cognition, using tests administered to the mother. This has received criticism as to its accuracy [43] and is mainly why we have preferred to use the level of education achieved which is likely to be more reflective of the individual’s IQ than the length of time in education used in some studies; (v) breast feeding was recorded at 6 months, and therefore is not affected by recall bias.

Among the limitations are those common to most longitudinal cohort studies, including attrition. We did not attempt to compensate for this since there was no evidence that the missing data were missing at random. However, several of the variables that were used as confounders were almost all associated with likelihood of attrition; consequently, by allowing for these statistically we probably have partly allowed for attrition. Recent analysis of attrition in other ALSPAC studies has shown minimal bias when using the complete case analysis as we have here [44].

An additional limitation is that the study population largely comprised parents who were of White European origin, which was true of the whole of the study area at the time that the study children were born (the early 1990s). Consequently, the results cannot be extrapolated worldwide or even to elsewhere in Britain or Europe.

A further limitation is the choice of confounders, which may not have been adequate or appropriate for each outcome. The choice of outcomes may also be criticised—by concentrating solely on conditions that are measured in ALSPAC on a continuous scale we have ignored non-linear associations as well as categorical ones. It should be noted also that this set of analyses is concentrated on neurocognitive outcomes, and therefore that conclusions in this group of outcomes regarding the benefit of breast feeding may not be appropriate for physiological outcomes such as obesity, asthma, eczema or allergies. These will be considered in further detailed sets of analyses.

By considering only those associations that passed stringent *p*-values, we are likely to have avoided Type I errors at the expense of including more Type II errors. In other words, failure to have found an adjusted association at *p* < 0.001 does not mean that there is no association.

## 5. Conclusions

These analyses confirm findings in the literature of the long-term intellectual benefits of breast feeding for 6 months on the development of the offspring, including their ability to exceed in academic tests of reading, math, and science. Although there is little sign of benefit to the child’s overall behaviour, or mental health, apart from a reduced risk of hyperactive behaviour in early childhood, there was an increased chance of being right-handed. There were no adverse neurocognitive effects detected. Consequently, we conclude that breast feeding, particularly for as long as 6 months, should be recommended for future neurocognitive benefits for the child. Causality cannot be inferred, and further studies are needed to confirm the results.

## Figures and Tables

**Table 1:** Unadjusted and adjusted comparisons of associations between children breast fed for 6 months with those never breast-fed concerning measures of child development (results at *p* < 0.001 are in bold).

Measure	UMD [95%CI] ^a^		AMD [95% CI] ^b^			AMD [95%CI] ^c^		
		*n*		*n*	*p*		*n*	*p*
**Griffiths Tests**								
At 18 months								
Social/personal	**+0.98 [0.52, 1.44]**	510	+0.59 [0.03, 1.15]	510	0.037	+0.57 [0.01, 1.13]	509	0.048
Hearing/speech	**+1.50 [0.81, 2.19]**	510	+0.90 [0.06, 1.73]	510	0.037	+0.87 [0.03, 1.72]	509	0.043
Total score	**+4.04 [2.18, 5.90]**	510	+2.37 [0.11, 4.64]	510	0.040	+2.27 [−0.01, 4.55]	509	0.051
**Denver Tests**								
Social Skills								
At 57 months	**−0.45 [−0.66, −0.24]**	4383						
Fine motor skills								
At 18 months	**+0.48 [0.32, 0.64]**	5450	+0.29 [0.09, 0.49]	4752	0.005	+0.28 [0.07, 0.48]	4740	0.007
At 30 months	**+0.71 [0.50, 0.91]**	5076	**+0.51 [0.25, 0.78]**	4464	**<0.001**	**+0.51 [0.25, 0.78]**	4452	**<0.001**
At 42 months	**+0.80 [0.57, 1.02]**	4961	**+0.50 [0.22, 0.78]**	4381	**0.001**	**+0.51 [0.22, 0.79]**	4368	**<0.001**
At 57 months	**+0.98 [0.81, 1.16]**	4377						
**Communication skills**								
At 57 months	**+0.31 [0.20, 0.42]**	4556	+0.05 [−0.09, 0.19]	4047	0.488	+0.05 [−0.09, 0.19]	4036	0.507
**Gross motor skills**								
At 18 months	**−0.40 [−0.56, −0.25]**	5447	−0.13 [−0.33, 0.06]	4749	0.171	−0.12 [−0.31, 0.07]	4732	0.219
At 30 months	**−0.31 [−0.44, −0.17]**	5068	−0.06 [−0.23, 0.11]	4457	0.490	−0.04 [−0.22, 0.13]	4445	0.609

^a^ *p* < 0.0001 for each measure; AMD = Adjusted mean difference; UMD = unadjusted mean difference; ^b^ adjusted for maternal education, paternal education, maternal age, whether firstborn, Caesarean birth, tenure of housing; ^c^ additionally adjusted for maternal smoking mid-pregnancy.

**Table 2:** Unadjusted and adjusted comparisons of associations between children breast fed for 6 months with those never breast-fed concerning components of measures of IQ (results at *p* < 0.001 are in bold).

Measure	UMD [95% CI] ^a^		AMD [95% CI] ^b^			AMD [95% CI] ^c^		
		*n*		*n*	*p*		*n*	*p*
**At age 4**								
Performance IQ	**+6.97 [4.28, 9.66]**	445	+2.40 [−0.67, +5.48]	446	0.125	+2.20 [−0.87, 5.27]	445	0.159
Verbal IQ	**+7.64 [5.07, 10.22]**	445	+3.32 [0.46, 6.19]	444	0.023	+3.36 [0.48, 6.23]	443	0.022
Full-Scale IQ	**+8.50 [5.81, 11.19]**	445	+3.48 [0.50, 6.45]	444	0.022	+3.40 [0.41, 6.38]	443	0.026
**At age 8**								
Performance IQ	**+7.79 [6.62, 8.96]**	3455	**+3.04 [1.65, 4.44]**	3110	****	**+3.00 [1.60, 4.40]**	3100	****
Verbal IQ	**+10.76 [9.62, 11.90]**	3460	**+4.13 [2.83, 5.44]**	3113	****	**+4.23 [2.92, 5.54]**	3103	****
Full scale IQ	**+10.60 [9.49, 11.72]**	3442	**+4.06 [2.79, 5.33]**	3098	****	**+4.11 [2.83, 5.39]**	3088	****
Verbal comprehension	**+7.20 [6.43, 7.97]**	3426	**+2.80 [1.92, 3.68]**	3084	****	**+2.85 [1.96, 3.73]**	3074	****
Perceptual organisation	**+5.13 [4.37, 5.89]**	3262	**+2.27 [1.37, 3.18]**	2937	****	**+2.26 [1.34, 1.17]**	2927	****
Freedom of distractibility	**+2.65 [2.23, 3.08]**	3353	+0.82 [0.32, 1.33]	3020	0.001	+0.85 [0.34, 1.36]	3010	0.001
**At age 15**								
Vocabulary	**+7.86 [6.90, 8.82]**	2493	**+3.38 [2.32, 4.43]**	2282	****	**+3.37 [2.31, 4.43]**	2279	****
Matrix reasoning	**+3.72 [3.01, 4.42]**	2492	**+1.71 [0.88, 2.54]**	2280	****	**+1.72 [0.89, 2.55]**	2277	****
Total IQ (sum of 2 tests)	**+9.02 [7.93, 10.10]**	2490	**+5.12 [3.57, 6.66]**	2279	****	**+5.12 [3.57, 6.67]**	2276	****

^a^ *p* < 0.0001 for each measure; **** = *p* < 0.0001; AMD = Adjusted mean difference; UMD = unadjusted mean difference; ^b^ adjusted for maternal education, paternal education, maternal age, whether firstborn, Caesarean birth, tenure of housing; ^c^ additionally adjusted for maternal smoking mid-pregnancy.

**Table 3:** Unadjusted and adjusted comparisons of associations between children breast fed for 6 months with those never breast fed concerning measurements of memory. (results at *p* < 0.001 are in bold).

Measure	UMD [95% CI] ^a^		AMD [95% CI] ^b^			AMD [95% CI] ^c^		
		*n*		*n*	*p*		*n*	*p*
**Digit span**								
At 8 years	**+1.02 [0.80, 1.24]**	3369	+0.27 [0.01, 0.54]	3034	0.043	+0.27 [0.00, 0.53]	3024	0.047
Forward at 8 years	**+0.30 [0.22, 0.37]**	3389	+0.08 [−0.01, 0.18]	3048	0.075	+0.08 [−0.01, 0.18]	3038	0.090
Backwards at 8 years	**+0.16 [0.10, 0.22]**	3375	+0.04 [−0.03, 0.11]	3040	0.310	+0.04 [−0.04, 0.11]	3030	0.310
**Working memory**								
At 8 years ^d^	**+1.02 [0.80, 1.24]**	3455	**+0.50 [0.30, 0.70]**	3111	**<0.001**	**+0.49 [0.29, 0.70]**	3102	**<0.001**
At 10 years	**+0.20 [0.14, 0.26]**	3286	+0.05 [−0.02, 0.13]	2956	0.181	+0.04 [−0.03, 0.12]	2946	0.244
At 9 years ^d^	**+0.83 [0.66, 1.01]**	6605	+0.98 [0.21, 1.75]	3358	0.012	+1.02 [0.25, 1.80]	3347	0.009
At 12 years ^d^	**+3.03 [1.69, 4.37]**	1651	+0.31 [−1.27, 1.90]	823	0.701	+0.44 [−1.14, 2.03]	820	0.585
At 13 years ^d^	**+3.42 [2.66, 4.19]**	4633	+1.32 [0.41, 2.23]	2386	0.004	+1.30 [0.39, 2.22]	2381	0.005

^a^ *p* < 0.0001 for each measure; AMD = Adjusted mean difference; UMD = unadjusted mean difference; ^b^ adjusted for maternal education, paternal education, maternal age, whether firstborn, Caesarean birth, tenure of housing; ^c^ additionally adjusted for smoking mid-pregnancy; ^d^ measured using non-word repetition.

**Table 4:** Unadjusted and adjusted comparisons of associations between children breast fed for 6 months with those never breast-fed concerning measures of speech and language (results at *p* < 0.001 are in bold).

Measure	UMD [95% CI] ^a^		AMD [95% CI] ^b^			AMD [95% CI] ^c^		
		*n*		*n*	*p*		*n*	*p*
**Reynell** At 25 months								
Verbal comprehension ^d^	**+4.68 [3.24, 6.12]**	538	+2.77 [1.06, 4.49]	491	0.002	+2.83 [1.10, 4.55]	490	0.001
At 61 months								
Incorrect alveolars d	**−0.60 [−0.88, −0.32]**	475	−0.25 [ −0.55, 0.06]	438	0.117	−2.30 [−0.54, 0.08]	437	0.148
**CCC at 9 years**								
Syntax score	**+0.11 [0.07, 0.14]**	3882	+0.04 [−0.00, 0.09]	3499	0.066	+0.04 [−0.01, 0.09]	3487	0.082
Inappropriate initiation	**+0.92 [0.77, 1.07]**	3878	**+0.51 [0.33, 0.70]**	3495	**<0.001**	**+0.50 [0.31, 0.69]**	3483	**<0.001**
Coherence	**+0.28 [0.15, 0.42]**	3882	−0.10 [−0.10, 0.24]	3498	0.402	+0.05 [−0.12, 0.22]	3486	0.554
Stereotyped conversation	**+0.85 [0.67, 0.99]**	3865	**+0.50 [0.30, 0.70]**	3487	**<0.001**	**+0.50 [0.30, 0.70]**	3487	**<0.001**
Use of conversation	**+0.85 [0.71, 0.99]**	3822	**+0.41 [0.24, 0.57]**	3451	**<0.001**	**+0.39 [0.23, 0.56]**	3439	**<0.001**
Pragmatic aspects of conversation score	**+2.99 [2.46, 3.51]**	3807	**+1.41 [0.77, 2.05]**	3437	**<0.001**	**+1.34 [0.70, 1.98]**	3425	**<0.001**
**WOLD ^d^ at 8 years**								
Comprehension	**+0.75 [0.61, 0.89]**	3457	+0.23 [0.07, 0.40]	3112	0.005	+0.23 [0.07, 0.40]	3103	0.006
**Mother at 24 months**								
Vocabulary	**+15.06 [11.99, 18.13]**	8814	**+8.19 [4.37, 12.01]**	4502	**<0.001**	**8.11 [4.27, 11.95]**	4487	**<0.001**
Grammar 1	**+0.36 [0.22, 0.51]**	8814	+0.16 [−0.02, 0.34]	4501	0.081	0.18 [−0.00, 0.36]	4487	0.056
Plurals	**+0.29 [0.15, 0.43]**	8814	+0.14 [−0.04, 0.32]	4502	0.122	+0.14 [−0.03,0.32]	4487	0.114
Grammar 2	**+1.81 [0.94, 2.67]**	8196	+1.20 [0.11, 2.29]	4185	0.031	1.20 [0.11, 2.30]	4173	0.032
**Mother at 38 months**								
Vocabulary	**+7.47 [5.69, 9.25]**	8700	**+4.64 [2.44, 6.84]**	4434	**<0.001**	**+4.71 [2.50, 6.92]**	4421	**<0.001**
Plurals	**+0.48 [0.35, 0.61]**	8619	**+0.29 [0.13, 0.45]**	4393	**<0.001**	**+0.28 [0.12, 0.44]**	4381	**<0.001**
Past tense	**+2.05 [1.46, 2.63]**	8578	**+1.46 [0.72, 2.21]**	4371	**<0.001**	**+1.50 [0.75, 2.24]**	4358	**<0.001**
Word combination	**+1.56 [1.28, 1.84]**	8566	**+0.73 [0.39, 1.07]**	4362	**<0.001**	**+0.77 [0.43, 1.11]**	4349	**<0.001**
Language	**+9.38 [7.31, 11.45]**	8410	**+6.16 [3.61, 8.71]**	4281	**<0.001**	**+6.21 [3.65, 8.78]**	4268	**<0.001**

^a^ *p* < 0.0001 for each measure; AMD = adjusted mean difference; UMD = unadjusted mean difference; ^b^ adjusted for maternal education, paternal education, maternal age, whether firstborn, Caesarean birth, tenure of housing; ^c^ additionally adjusted for maternal smoking mid-pregnancy; ^d^ tested by trained psychologists.

**Table 5:** Unadjusted and adjusted measures of associations between breast feeding for 6 months and academic results in reading, spelling and other English language. (results at *p* < 0.001 are in bold).

Measure	UMD [95% CI] ^a^		AMD [95% CI] ^b^			AMD [95% CI] ^c^		
		*n*		*n*	*p*		*n*	*p*
**Reading** ^d^								
Reading at 7	**+4.38 [3.75, 5.00]**	6619	**+1.82 [1.08, 2.56]**	3366	**<0.001**	**+1.54 [1.08, 2.57]**	3555	**<0.001**
Word Reading at 9	**+1.08 [0.91, 1.25]**	6257	**+0.36 [0.16, 0.56]**	3186	**<0.001**	**+0.34 [0.14, 0.54]**	3176	**<0.001**
Comprehension at 9	**+7.05 [6.21, 7.89]**	5672	**+2.03 [1.07, 3.00]**	2899	**<0.001**	**+1.97 [1.00, 2.94]**	2890	**<0.001**
Speed at 9	**+6.43 [5.55, 7.32]**	5661	**+2.23 [1.19, 3.26]**	2891	**<0.001**	**+2.23 [1.18, 3.27]**	2882	**<0.001**
Accuracy at 9	**+6.98 [6.03, 7.93]**	5672	**+2.27 [1.15, 3.39]**	2899	**<0.001**	**+2.23 [1.10, 3.35]**	2890	**<0.001**
Fluency at TF1	**+3.23 [1.75, 4.72]**	1653	−0.20 [−1.93, 1.53]	824	0.819	−0.04 [−1.77, 1.69]	821	0.967
Fluency at TF2	**+4.00 [3.17, 4.84]**	4644	+1.27 [0.28, 2.25]	2391	0.012	+1.27 [0.29, 2.26]	2386	0.011
Phoneme deletion at 7	**+3.00 [2.36, 3.64]**	6605	+0.98 [0.21, 1.75]	3358	0.012	+1.02 [0.25, 1.80]	3347	0.009
**National Reading Tests**								
Entry assessment ^e^	**+0.42 [0.37, 0.47]**	6921	**+0.17 [0.10, 0.23]**	2386	**<0.001**	**+0.16 [0.10, 0.23]**	3344	**<0.001**
SATS 3 reading	**+4.23 [3.87, 4.60]**	7639	**+1.63 [1.20, 2.06]**	3735	**<0.001**	**+1.59 [1.16, 2.02]**	3722	**<0.001**
SATS 3 Shakespeare	**+1.99 [1.78, 2.19]**	7565	**+0.79 [0.54, 1.03]**	3695	**<0.001**	**+0.77 [0.52, 1.01]**	3685	**<0.001**
Self-perception of reading at 9	**+1.30 [0.97, 1.62]**	3461	+0.21 [−0.18, 0.60]	3127	0.290	+0.22 [−0.17, 0.62]	3117	0.265
**Spelling ^d^**								
At 7	**+1.37 [1.07, 1.67]**	6522	+0.31 [−0.05, 0.67]	3311	0.089	+0.31 [−0.05, 0.67]	3300	0.089
At 9	**+1.24 [1.00, 1.47]**	6238	+0.37 [0.09, 0.65]	3177	0.010	+0.35 [0.07, 0.63]	3167	0016
**National Spelling Test**								
KS2	**+1.13 [0.82, 1.44]**	1783	+0.28 [−0.12, 0.67]	852	0.167	0.30 [−0.10, 0.69]	849	0.139
**English Language**	**+0.36 [0.29, 0.42]**	6920	+0.68 [−0.01, 0.15]	3352	0.107	+0.07 [−0.02, 0.15]	3342	0.121
SATS 3	**+11.89 [10.89, 12.88]**	7559	**+4.45 [3.29, 5.60]**	3699	**<0.001**	**+4.30 [3.14, 5.46]**	3688	**<0.001**
**Writing ability**								
-school entry ^e^	**+0.28 [0.22, 0.33]**	6922	+0.07 [−0.00, 0.13]	3353	0.050	+0.06 [−0.00, 0.13]	3343	0.068
SATS3	**+5.66 [5.11, 6.21]**	7605	**+2.03 [1.37, 2.68]**	3721	**<0.001**	**+1.95 [1.29, 2.61]**	3710	**<0.001**

^a^ *p* < 0.0001 for each measure; AMD = Adjusted mean difference; UMD = unadjusted mean difference; ^b^ adjusted for maternal education, paternal education, maternal age, whether firstborn, Caesarean birth, tenure of housing; ^c^ additionally adjusted for maternal smoking mid-pregnancy; ^d^ tested by ALSPAC staff; ^e^ tested by teacher at school entry.

**Table 6:** Unadjusted and adjusted measures of associations between breast feeding for 6 months and Mathematics and Science test results (results at *p* < 0.001 are in bold).

Measure	UMD [95% CI] ^a^		AMD [95% CI] ^b^			AMD [95%CI] ^c^		
		*n*		*n*	*p*		*n*	*p*
Arithmetic								
At 4 years	**+1.31 [0.82, 1.80]**	487	+0.38 [−0.17, 0.92]	446	0.172	+0.39 [−0.16, 0.94]	4450	0.163
At 8 years	**+1.24 [1.00, 1.48]**	3431	+0.54 [0.20, 0.88]	3116	0.002	+0.57 [0.22, 0.91]	3106	0.001
**Maths Comprehension**								
At 8 years	**+1.56 [1.31, 1.81]**	3854	+0.43 [0.12, 0.75]	1936	0.006	+0.42 [0.10, 0.73]	1931	0.009
At 10 years	**+4.06 [3.60, 4.52]**	5807	**+1.56 [1.00, 2.13]**	2902	**<0.001**	**+1.55 [0.98, 2.12]**	2895	**<0.001**
At 12 years	**+4.25 [3.48, 5.02]**	2113	+1.37 [0.45, 2.29]	1073	0.004	+1.41 [0.43, 2.33]	1071	0.003
**National Maths Tests**								
SATS2	**+12.34 [11.21, 13.46]**	8806	**+3.22 [1.89, 4.55]**	4362	**<0.001**	**+3.26 [1.92, 4.59]**	4349	**<0.001**
SATS3	**+12.42 [11.18, 13.67]**	7729	**+5.29 [3.77, 6.81]**	3779	**<0.001**	**+5.30 [3.77, 6.83]**	3768	**<0.001**
**Science**								
Reasoning	**+1.30 [1.13, 1.47]**	5819	+0.34 [0.14, 0.55]	2902	0.001	+0.33 [0.13, 0.54]	2896	0.002
Comprehension	**+0.33 [0.28, 0.38]**	5813	+0.11 [−0.22, 0.45]	2900	0.511	+0.14 [−0.20, 0.48]	2894	0.418

^a^ *p* < 0.0001 for each measure; AMD = adjusted mean difference; UMD = unadjusted mean difference; ^b^ adjusted for maternal education, paternal education, maternal age, whether firstborn, Caesarean birth, tenure of housing; ^c^ additionally adjusted for maternal smoking mid-pregnancy.

**Table 7:** Unadjusted and adjusted measures of associations between breast feeding for 6 months and the child’s temperament (results at *p* < 0.001 are in bold).

Measure	UMD [95% CI] ^a^		AMD [95% CI] ^b^			AMD [95% CI] ^c^		
		*n*		*n*	*p*		*n*	*p*
**Carey Scores at 24 months**								
Rhythmicity	**−0.69 [−1.01, −0.37]**	8838	+0.12 [−0.29, 0.53]	4524	0.566	+0.11 [−0.30, 0.52]	4509	0.606
Approach	**−1.04 [−1.46, −0.61]**	8839	−0.38 [−0.92, 0.16]	4526	0.168	−0.38 [−0.92, 0.17]	4511	0.173
Difficult temperament	**−2.36 [−3.31, −1.40]**	8798	−1.40 [−2.62, −0.18]	4502	0.025	−1.29 [−2.51, −0.06]	4487	0.040
**EAS scores**								
Activity at 38 months	**−0.74 [−0.92, −0.56]**	8655	**−0.54 [−0.77, −0.32]**	4411	**<0.001**	**−0.52 [−0.75, −0.30**]	4398	**<0.001**
Emotionality at 4 years	**+0.43 [0.23, 0.63]**	8160	+0.21 [−0.04, 0.45]	4155	0.097	+0.20 [−0.05, 0.45]	4144	0.110
Activity at 4 years	**−1.03 [−1.20, −0.86]**	8161	**−0.71 [−0.92, −0.49]**	4156	**<0.001**	**−0.69 [−0.90, −0.47]**	4145	**<0.001**
Emotionality at 5 years	**+0.43 [0.22, 0.64]**	7550	+0.19 [−0.06, 0.45]	3878	0.137	+0.19 [−0.07, 0.44]	3866	0.153
Activity at 5 years	**−0.97 [−1.15, −0.79]**	7549	**−0.63 [−0.85, −0.40]**	3879	**<0.001**	**−0.62 [−0.84, −0.39]**	3867	**<0.001**

^a^ *p* < 0.0001 for each measure; AMD = Adjusted mean difference; UMD = unadjusted mean difference; ^b^ adjusted for maternal education, paternal education, maternal age, whether firstborn, Caesarean birth, tenure of housing; ^c^ additionally adjusted for maternal smoking mid-pregnancy.

**Table 8:** Unadjusted and adjusted measures of associations between breast feeding for 6 months and the child’s behaviour (results at *p* < 0.001 are in bold).

Measure	UMD [95% CI] ^a^		AMD [95% CI] ^b^			AMD [95% CI] ^c^		
		*n*		*n*	*p*		*n*	*p*
**SDQ**								
Prosocial at 9 years ^d,e^	**−0.31 [−0.42, −0.20]**	6789	**−0.24 [−0.38, −0.10]**	3485	**<0.001**	**−0.24 [−0.38, −0.11]**	3473	**<0.001**
Hyperactivity at 47 months ^d^	**−0.80 [−0.93, −0.66]**	8266	**−0.36 [−0.53, −0.19]**	4202	**<0.001**	**−0.35 [−0.52, 0.18]**	4189	**<0.001**
Hyperactivity at 6 years ^d^	**−0.61 [−0.75, −0.46]**	7385	−0.26 [−0.44, −0.08]	3824	0.005	−0.23 [−0.41, −0.05]	3811	0.011
Hyperactivity at 9 years ^d^	**−0.40 [−0.54, −0.25]**	6787	−0.06 [−0.24, 0.12]	3483	0.484	−0.04 [−0.22, 0.14]	3471	0.683
Hyperactivity at 11 years ^d^	**−0.46 [−0.61, −0.30]**	6226	−0.21 [−0.39, −0.02]	3198	0.030	−0.19 [−0.38, −0.01]	3187	0.042
Hyperactivity at 7 years ^f^	**−0.82 [−1.02, −0.62]**	4718	−0.30 [−0.55, −0.05]	2321	0.017	−0.27 [−0.51, −0.01]	2314	0.033
Hyperactivity at 10 years ^f^	**−0.71 [−0.88, −0.53]**	5397	−0.12 [−0.34, 0.10]	2669	0.287	−0.10 [−0.33, 0.12]	2661	0.362
**Emotional symptoms**								
At 10 years ^f^	**−0.27 [−0.40, −0.14]**	5397	−0.05 [−0.21, 0.11]	2669	0.543	−0.05 [−0.21, 0.12]	2661	0.583
**Conduct problems**								
At 47 months ^d^	**−0.19 [−0.27, −0.12]**	8266	−0.01 [−0.11, 0.09]	4202	0.853	+0.01 [−0.09, 0.11]	4189	0.777
At 7 years ^f^	**−0.34 [−0.44, −0.24]**	4715	−0.09 [−0.21, 0.04]	2318	0.173	−0.09 [−0.21, 0.04]	2318	0.173
At 10 years ^f^	**−0.31 [−0.42, −0.20]**	5395	−0.05 [−0.17, 0.08]	2669	0.471	−0.07 [−0.19, 0.06]	2311	0.289
**Peer difficulties**								
At 47 months ^d^	**−0.26 [−0.35, −0.18]**	8266	−0.10 [−0.21, 0.01]	4202	0.076	−0.09 [−0.20,0.02]	4189	0.094
**Total difficulties**								
At 47 months ^d^	**−1.30 [−1.55, −1.04]**	8266	−0.50 [−0.82, 0.17]	4202	0.003	−0.46 [−0.79, −0.14]	4189	0.005
At 6 years ^d^	**−0.70 [−0.99, −0.41]**	7377	−0.07 [−0.43, 0.29]	3822	0.691	−0.01 [−0.37, 0.35]	3809	0.950
At 9 years ^d^	**−0.68 [−1.00, −0.36]**	6763	−0.03 [0.43, 0.36]	3473	0.866	+0.02 [−0.37, 0.42]	3461	0.908
At 11 years ^d^	**−0.84 [−1.17, −0.50]**	6236	−0.29 [−0.10, 0.12]	3202	0.163	−0.28 [−0.69, 0.13]	3191	0.177
At 7 years ^f^	**−1.54 [−1.95, −1.12]**	4718	−0.64 [−1.16, −0.13]	2321	0.014	−0.58 [1.09, −0.07]	2314	0.027
At 10 years ^f^	**−1.34 [−1.74, −0.95]**	5397	−0.14 [−0.61, 0.34]	2669	0.579	−0.09 [−0.57, 0.39]	2661	0.711
**DAWBA at 91 months ^d^**								
No. general anxiety symptoms	**+0.21 [0.11, 0.30]**	7070	0.00 [−0.11, 0.12]	3665	0.942	+0.01 [−0.11, 0.12]	3653	0.923
No. activity symptoms	**−0.47 [−0.64, −0.31]**	1108	−0.20 [−0.41, 0.01]	3684	0.056	−0.19 [−0.40, 0.02]	3672	0.077
Activity symptoms score	**−0.61 [−0.84, −0.39]**	7083	−0.26 [−0.54, 0.02]	3673	0.069	−0.24 [−0.52, 0.04]	3662	0.087
Total no. attention/activity symptoms	**−0.70 [−1.01, −0.39]**	7116	−0.24 [−0.63, 0.15]	3690	0.221	−0.20 [−0.59, 0.18]	3678	0.301
Attention/activity score	**−0.93 [−1.36, −0.50]**	7096	−0.33 [−0.85, 0.20]	3682	0.221	−0.29 [−0.82, 0.24]	3670	0.286

^a^ *p* < 0.0001 for each measure; AMD = adjusted mean difference; UMD = unadjusted mean difference; ^b^ Adjusted for maternal education, paternal education, maternal age, whether firstborn, Caesarean birth, tenure of housing; ^c^ additionally adjusted for maternal smoking mid-pregnancy; ^d^ completed by the mother; ^e^ Negative mean score indicates that the breast-fed children are more social; ^f^ completed by the child’s teacher.

**Table 9:** Unadjusted and adjusted measures of associations between breast feeding for 6 months and other attributes of the child (results at *p* < 0.001 are in bold).

Measure	UMD [95% CI] ^a^		AMD [95% CI] ^b^			AMD [95% CI] ^c^		
		*n*		*n*	*p*		*n*	*p*
**Locus of control**								
At 8 years	**−0.87 [−1.02, −0.71]**	5286	**−0.33 [−0.52, −0.15]**	2684	**<0.001**	**−0.33 [−0.51, −0.14]**	2674	**<0.001**
At 16 years	**+0.44 [0.26, 0.62]**	4037	−0.01 [−0.22, 0.20]	2124	0.940	−0.01 [−0.22, 0.20]	2119	0.931
Self-esteem at 9 years	**−3.58 [−4.93, −2.23]**	4624	−2.90 [−4.56, −1.24]	2353	0.001	−2.93 [−4.59, −1.26]	2324	0.001
**Cognitive style**								
Complete item total	**+4.56 [2.33, 6.79]**	2818	+3.46 [0.79, 6.13]	1484	0.011	+3.61 [0.93, 6.29]	1478	0.008
Achievement events	**+2.89 [1.94, 3.85]**	2818	+1.94 [0.81, 3.08]	1484	0.001	+ 2.00 [0.87, 3.14]	1478	0.001
**Personality at 13 years**								
Agreeable	**+1.25 [0.84, 1.66]**	4802	+0.13 [−0.36, 0.63]	1460	0.595	+0.16 [−0.34, 0.65]	2454	0.527
Intellectual	**+1.96 [1.51, 2.42]**	4780	+0.74 [0.20, 1.27]	2459	0.007	+0.79 [0.25, 1.33]	2453	0.004
Sensation seeking at 18	**+2.22 [1.42, 3.01]**	2805	+1.08 [0.14, 2.02]	1481	0.025	+1.10 [0.15, 2.04]	1475	0.023
**Motor coordination**								
Handedness at 42 months	**+0.09 [0.06, 0.12]**	8584	**+0.08 [0.04, 0.12]**	4364	**<0.001**	**+0.08 [0.04, 0.12]**	4351	**<0.001**
Heel to toe at 7 years	**+0.41 [0.26, 0.57]**	5922	+0.23 [0.06, 0.41]	3026	0.010	+0.24 [0.06, 0.42]	3018	0.009
Standing on R leg, eyes open at 10 years	**+0.65 [0.37, 0.93]**	6010	+0.44 [0.10, 0.78]	3079	0.012	+0.46 [0.11, 0.80]	3069	0.009
Balance score at 10 years	**−0.66 [−0.93, −0.40]**	5887	−0.30 [−0.62, 0.02]	3023	0.064	−0.29 [−0.61, 0.03]	3013	0.078
**Motor task**								
Time at 8 years	**−1.76 [−2.35, −1.16]**	6032	−1.20 [1.94, −0.46]	3090	0.001	−1.17 [−1.91, −0.43]	3080	0.002
Task at 8 years	**−0.10 [−0.13, −0.07]**	5982	−0.07 [−0.11, −0.03]	3064	0.001	−0.06 [−0.10, −0.02]	3054	0.001
Time at 11 years	**−1.09 [−1.54, −0.64]**	5826	−0.60 [−1.15, −0.05]	2950	0.034	−0.63 [−1.18, −0.07]	2941	0.027
Task at 11 years	**−0.06 [−0.08, −0.03]**	5806	−0.04 [−0.07, −0.01]	2943	0.021	−0.04 [−0.07, −0.01]	2934	0.016
Opposite world time at 11 years	**−0.39 [−0.58, −0.21]**	5592	+0.02 [−0.20, 0.24]	2825	0.833	+0.03 [−0.19, 0.25]	2816	0.806
**Executive function**								
Inhibition 1st block reaction time	**−4.60 [−6.89, −2.32]**	5741	−1.77 [−4.60, 1.05]	2944	0.218	−1.78 [−4.62, 1.06]	2934	0.220
**Addictions**								
Alcohol								
No. drinks to feel tipsy when starting to drink	**−0.45 [−0.63, −0.27]**	2801	−0.25 [−0.47, −0.03]	1471	0.024	−0.25 [−0.47, −0.03]	1466	0.023
Current no. drinks at 20 years	**−0.59 [−0.86, −0.31]**	2916	−0.36 [−0.69, −0.03]	1520	0.031	−0.36 [−0.69, −0.03]	1516	0.035
AUDIT score	**+1.17 [0.64, 1.70]**	3346	+0.63 [−0.01, 1.28]	1739	0.054	+0.64 [−0.01, 1.28]	1734	0.053

^a^ *p* < 0.0001 for each measure; AMD = Adjusted mean difference; UMD = unadjusted mean difference; ^b^ adjusted for maternal education, paternal education, maternal age, whether firstborn, Caesarean birth, tenure of housing; ^c^ additionally adjusted for maternal smoking mid-pregnancy.

## Data Availability

ALSPAC data is available to researchers for particular projects, provided no attempt is made to reveal the identities of the subjects. Guidelines for access are found on the ALSPAC website: www.bristol.ac.uk/alspac/researchers.

## References

[1] Kramer M.S. (2010). “Breast is best”: The evidence. Early Hum. Dev..

[2] Yang S., Martin R.M., Oken E., Hameza M., Doniger G., Amit S., Patel R., Thompson J., Rifas-Shiman S.L., Vilchuck K. (2018). Breastfeeding during infancy and neurocognitive function in adolescence: 16-year follow-up of the PROBIT cluster-randomized trial. PLoS Med..

[3] Kramer M.S., Fombonne E., Matush L., Bogdanovich N., Dahhou M., Platt R.W. (2011). Long-term behavioural consequences of infant feeding: The limits of observational studies. Paediatr. Perinat. Epidemiol..

[4] Horta B.L., Victora C.G. (2013). Long-Term Effects of Breastfeeding.

[5] McGowan C., Bland R. (2023). The benefits of breastfeeding on child intelligence, behavior, and executive function: A review of recent evidence. Breastfeed. Med..

[6] Hou L., Li X., Yan P., Li Y., Wu Y., Yang Q., Shi X., Ge L., Yang K. (2021). Impact of the duration of breastfeeding on the intelligence of children: A systematic review with network meta-analysis. Breastfeed. Med..

[7] Adams L.J., Pell J.P., Mackay D.F., Clark D., King A., Fleming M. (2023). Infant feeding method and special educational need in 191,745 Scottish schoolchildren: A national, population cohort study. PLoS Med..

[8] Sanefuji M., Senju A., Shimono M., Ogawa M., Sonoda Y., Torio M., Ichimiya Y., Suga R., Sakai Y., Honjo S. (2021). Breast feeding and infant development in a cohort with sibling pair analysis: The Japan Environment and Children’s Study. BMJ Open.

[9] Lovcevic I. (2023). Associations of breastfeeding duration and cognitive development from childhood to middle adolescence. Acta Paediatr..

[10] Golding J., Pembrey M., Jones R., The ALSPAC Study Team (2001). ALSPAC–The Avon Longitudinal Study of Parents and Children. Paediatr. Perinat. Epidemiol..

[11] Boyd A., Golding J., Macleod J., Lawlor D.A., Fraser A., Henderson J., Molloy L., Davey Smith G. (2013). Cohort profile: The ‘children of the 90s’—The index offspring of the Avon Longitudinal Study of Parents and Children. Int. J. Epidemiol..

[12] Fraser A., Macdonald-Wallis C., Tilling K., Boyd A., Golding J., Davey Smith G., Henderson J., Lawlor D.A. (2013). Cohort profile: The Avon Longitudinal Study of Parents and Children: ALSPAC mothers cohort. Int. J. Epidemiol..

[13] Birmingham K. (2018). Pioneering Ethics in Longitudinal Study: The Early Development of the ALSPAC Ethics & Law Committee.

[14] Zanardo V., Svegliado G., Cavallin F., Giustardi A., Cosmi E., Litta P., Trevisanuto D. (2010). Elective cesarean delivery: Does it have a negative effect on breastfeeding?. Birth.

[15] Correa M.L., Soares P.S.M., da Silva B.G.C., Wehrmeister F., Horta B.L., Menezes A.M.B. (2021). Maternal smoking during pregnancy and intelligence quotient in offspring: A systematic review and meta-analysis. Neurotoxicology.

[16] Napierala M., Mazela J., Merritt T.A., Florek E. (2016). Tobacco smoking and breastfeeding: Effect on the lactation process, breast milk composition and infant development. A critical review. Environ. Res..

[17] Griffiths R.J. (1970). The Abilities of Babies: A Study in Mental Measurement.

[18] Frankenburg W.K., Dodds J.B. (1967). Denver Developmental Screening Test. J. Pediatr..

[19] Wechsler D. (1990). Wechsler Preschool and Primary Scale of Intelligence–(RevisedUK Ed.).

[20] Wechsler D., Golombok S., Rust J. (1992). WISC-CN2IUK Wechsler Intelligence Scale for Children—(3rd Ed. UK Manual).

[21] Wechsler D. (2011). Wechsler Abbreviated Scale of Intelligence (2nd Ed. WASI-II).

[22] Gathercole S.E., Willis C.S., Baddeley A.D., Emslie H. (1994). The children’s test of nonword repetition: A test of phonological working memory. Memory.

[23] Case R., Kurland D.M., Goldberg J. (1982). Operational efficiency and the growth of short-term memory span. J. Exp. Child Psychol..

[24] Reynell J.K. (1978). Manual for the Reynell Developmental Language Scales (Revised).

[25] Rust R. (1996). Weschler Objective Language Dimensions Manual.

[26] Bishop D.V.M. (1998). Development of the Children’s Communication Checklist (CCC): A method for assessing qualitative aspects of communicative impairment in children. J. Child Psychol. Psychiatry.

[27] Golding J., Gregory S., Clark R., Iles-Caven Y., Ellis G., Taylor C.M., Hibbeln J. (2021). Maternal prenatal vitamin B12 intake is associated with speech development and mathematical abilities in childhood. Nutr. Res..

[28] Carey W.B., McDevitt S.C. (1978). Revision of the Infant Temperament Questionnaire. Pediatrics.

[29] Fullard W., McDevitt S.C., Carey W.B. (1978). Toddler Temperament Scale (1–3-Year-Old Children).

[30] Buss A.H., Plomin R. (1984). Temperament: Early Developing Personality Traits.

[31] Goodman R. (1997). The strengths and difficulties questionnaire: A research note. J. Child Psychol. Psychiatry Allied Discip..

[32] Goodman R., Ford T., Richards H., Gatward R., Meltzer H. (2000). The development and well-being assessment: Description and initial validation of an integrated assessment of child and adolescent psychopathology. J. Child Psychol. Psychiatry.

[33] Nowicki S., Iles-Caven Y., Gregory S., Ellis G., Golding J. (2018). Stability of, and associations between, parent and child locus of control expectancies. Front. Psychol..

[34] Goldberg L.R. (1992). The development of markers for the Big-Five factor structure. Psychol. Assess..

[35] Meins E., McCarthy-Jones S., Fernyhough C., Lewis G., Bentall R.P., Alloy L.B. (2012). Assessing negative cognitive style: Development and validation of a Short-Form version of the Cognitive Style Questionnaire. Personal. Individ. Differ..

[36] Zeng Y., Tang Y., Tang J., Shi J., Zhang L., Zhu T., Xiao D., Qu Y., Mu D. (2020). Association between the different duration of breastfeeding and attention deficit/hyperactivity disorder in children: A systematic review and meta-analysis. Nutr. Neurosci..

[37] Denny K. (2012). Breastfeeding predicts handedness. Laterality Asymmetries Body Brain Cogn..

[38] Hujoel P.P. (2019). Breastfeeding and handedness: A systematic review and meta-analysis of individual participant data. Laterality Asymmetries Body Brain Cogn..

[39] Pang W.W., Tan P.T., Cai S., Fok D., Chua M.C., Lim S.B., Shek L.P., Chan S.Y., Tan K.H., Yap F. (2020). Nutrients or nursing? Understanding how breast milk feeding affects child cognition. Eur. J. Nutr..

[40] Lockyer F., McCann S., Moore S.E. (2021). Breast milk micronutrients and infant neurodevelopmental outcomes: A systematic review. Nutrients.

[41] Dror D.K., Allen L.H. (2018). Retinol-to-fat ratio and retinol concentration in human milk show similar time trends and associations with maternal factors at the population level: A systematic review and meta-analysis. Adv. Nutr..

[42] Spiller P., Brenna J.T., Carlson S.E., Golding J., Crawford M.A., Hibbeln J.R., Koletzko B.V., Columbo J., Kris-Etherton P., Connor S.L. (2025). Fish consumption advice is depriving children of neurolipids and other nutrients essential to brain and eye development. NeuroToxicology.

[43] Sorjonen K., Nilsonne G., Ingre M., Melin B. (2024). Breastfeeding, cognitive ability, and residual confounding: A comment on studies by Pereyra-Elìas et al. PLoS ONE.

[44] Morgan J., Major-Smith D. (2024). Quantifying potential selection bias in observational research: Simulations and Analyses exploring religion and depression using a prospective UK cohort study (ALSPAC). Relig. Brain Behav..

